# Periodontal Nonsurgical Therapy With the Adjunctive Use of a Local Antibiotic for the Treatment of Deep Infrabony Defects in the Aesthetic Area: A Case Series

**DOI:** 10.1155/crid/6761897

**Published:** 2026-07-31

**Authors:** Ilham Mounssif, Diego Bianchelli, Claudio Mazzotti, Valentina Bentivogli, Anna Skurska, Martina Stefanini

**Affiliations:** ^1^ Department of Biomedical and Neuromotor Sciences (DIBINEM), Periodontology Unit, Alma Mater Studiorum—University of Bologna, Bologna, Italy; ^2^ Department of Integrated Dentistry, Medical University of Białystok, Białystok, Poland, umb.edu.pl

**Keywords:** doxycycline, infrabony defects, local antibiotic, minimally invasive approach, nonsurgical therapy, regenerative periodontal surgery

## Abstract

**Background/Objectives:**

The presence of a vertical bone defect in an aesthetic area is common among many patients. The reduction of supracrestal soft tissue often occurs alongside the recession of interdental papillae, particularly following nonsurgical therapy. This case series study is aimed at presenting a clinical approach that combines ultrasound and localized antibiotics for managing deep infrabony defects in aesthetic zones. The goal is to achieve bleeding cessation and minimize recession prior to predictable regenerative periodontal surgery.

**Methods:**

Ten patients diagnosed with periodontitis: Each subject had at least one interproximal site in the aesthetic area presenting a PD value > 5 mm and BOP+ associated with a defect with radiographic infrabony component > 3 mm. FMPS, FMBS, PPD, REC, CAL, and BOP were performed at baseline and at 3 months. Conventional periodontal nonsurgical therapy was performed in all sites to achieve FMPS and FMBS values below 20%, whereas the infrabony defect site received a tailored treatment as follows: Ultrasonic tips were used incrementally (Tip A up to 4‐mm subgingival, Tip P up to 7 mm, and Tip PS up to 11 mm) and a final application of 14% doxycycline‐based antibiotic delivered by a controlled‐release biodegradable vector.

**Results:**

All patients reported an improvement in PD values with no increase in REC and showed a negative BOP value. Mean PD decreased from 7.3 ± 1.9 mm to 5.2 ± 1.3 mm (mean reduction: 2.1 mm). Mean REC decreased from 0.9 ± 0.9 mm at baseline to 0.4 ± 0.5 mm at 3 months. BOP resolved in all treated sites (100%). No increase in REC was recorded in any patient.

**Conclusions:**

Nonsurgical therapy utilizing a minimally invasive approach with incremental ultrasonic tips and a local 14% doxycycline antibiotic seems capable of reducing inflammation parameters such as PPD and BOP without causing increased recession to enable predictable regenerative surgery.

## 1. Introduction

The ultimate goal of periodontal therapy is to regenerate the supporting tissue of the tooth that has been destroyed by periodontal disease; this regeneration is characterized by the formation of new root cementum, into which new collagen fibers are inserted, along with new periodontal ligament and new alveolar bone [1].

Under ideal conditions, following regenerative therapy of infrabony defects, the gain in clinical attachment level (CAL) should equal the reduction in pocket depth ( pocket depth [PD]); in fact, gingival recession (REC) should not increase as a result of the treatments performed [2–5].

The primary goals of nonsurgical and surgical periodontal therapy are disease control, reduction of inflammation, PD reduction, and clinical attachment gain. In addition, and particularly in aesthetically sensitive areas, minimizing gingival REC and maintaining the integrity of interdental soft tissue represent important additional, esthetically relevant treatment goals. Meeting these objectives allows increasing the potential of both the CAL and the bone level, as well as achieving satisfactory aesthetic results. By these considerations, the objectives of regenerative therapy in aesthetic areas encompass more than just completely filling the infrabony defect and achieving an increase in CAL. It is also important to maintain or even enhance the aesthetic appearance of the soft tissue.

In cases of a deep vertical bone defect, whether on the buccal side or in the interdental areas, the volume and thickness of the soft tissue covering the infrabony defect (supracrestal soft tissue [SCST]) are often maintained or even increased, even in the absence of REC. This preservation or increase of soft tissues is frequently due to the increased interdental space [4, 6, 7].

During regenerative surgery, the SCST is separated from the granulation tissue and used to cover the infrabony defect. Having a thick, high, and wide SCST facilitates flap management and the surgical suturing technique. Additionally, it enhances the likelihood of achieving and maintaining a primary closure in the interdental areas. Ultimately, it reduces the risk of the soft tissue collapsing within the bone defect. All this optimizes the possibility of the regeneration of the defect [3, 8]. Preserving SCST has proven to be important, especially when using membranes and enamel matrix derivatives (EMD) [4–7, 9, 10]. Recent data also indicate a positive and significant correlation between CALs, bone level gain, and the amount of SCST present [6, 7, 9–11].

The amount of SCST may decrease if the interdental papilla has receded, especially as a result of periodontal nonsurgical therapy and in particular following root‐planing procedures. Trauma to the buccal soft tissue may lead to the occurrence of gingival REC and consequently loss of vestibular keratinized tissue. From a surgical standpoint, the presence and quality of vestibular keratinized tissue are crucial factors influencing the outcome of the surgical procedure itself, as the loss of SCST and the loss of vestibular keratinized tissue before surgery could jeopardize the predictability of the regenerative surgical procedure.

According to the latest EFP guidelines for the treatment of Stage I–III periodontitis [11] nonsurgical therapy outcomes can be enhanced with the use of locally adjunctives administered such as chlorhexidine and antibiotics that may be considered to reduce PD in the short and long‐term follow‐up with minor adverse events reported [12]. Among antibiotics, Ligosan was investigated in three randomized controlled trial (RCT) supporting its benefits (PD reduction and clinical attachment level gain [CAL gain]) with respect to a control group in association with subgingival hand or ultrasonic instrumentations [13–15]. This product is a biodegradable highly viscous gel for topical subgingival placement composed of a carrier gel (polyethylene glycol lactide/glycolide copolymers) and the active ingredient doxycycline hyclate (16% of the finished formulation, which is equivalent to 14% doxycycline base, doxycycline free amine, respectively) (*Ligosan, Slow Release; Heraeus Kulzer GmbH*). Beyond its antimicrobial activity, doxycycline inhibits matrix metalloproteinases (MMPs)—particularly MMP‐8 and MMP‐9, which are major mediators of connective tissue breakdown in periodontal disease [16, 17]. This MMP‐inhibitory property may limit apical migration of the junctional epithelium and reduce extracellular matrix degradation during the early healing phase, potentially contributing to the preservation of the SCST architecture. Furthermore, doxycycline′s anti‐inflammatory properties may attenuate the postinstrumentation inflammatory response, reducing the risk of soft tissue shrinkage associated with conventional nonsurgical therapy. It should be emphasized that these represent biologically plausible hypotheses based on existing pharmacological evidence.

This study is aimed at presenting a series of clinical cases to show a minimally invasive, nonsurgical therapy for treating deep infrabony defects in aesthetic areas. This approach combines the use of ultrasonic tips with locally administered antibiotics to eliminate bleeding on probing (BOP) and reduce the risk of increased gingival REC. The primary hypothesis is that this combined protocol can achieve adequate PD reduction and BOP resolution while preserving SCST integrity, thereby optimizing conditions for subsequent regenerative surgery in aesthetically sensitive sites.

## 2. Case Presentation

### 2.1. Inclusion and Exclusion Criteria

**Figure 1:**
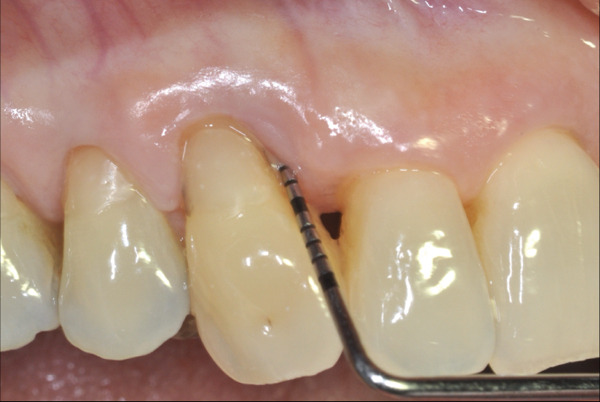
Buccal aspect at the level of the site with an infraosseous defect at baseline.

**Figure 2:**
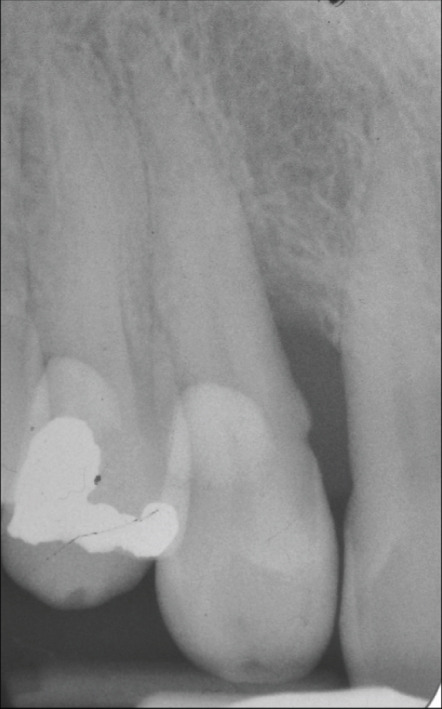
Radiograph at the level of the site with an infraosseous defect at baseline.

Ten patients referred to the Periodontology Department of the University of Bologna, diagnosed with periodontitis (Stages II–IV) [18], who had the following characteristics, were included in the study (Figures 1 and 2):

• At least one interproximal site in the esthetic zone (premolar to premolar) characterized by a PD value > 5 mm.
• Presence of bleeding on probing (BOP+) associated with a bone defect with radiographic infraosseous component > 3 mm [19].
• Age ≥ 18 years.

The following exclusion criteria were also taken into account:

• Systemic pathologies (e.g., diabetes mellitus, immunosuppressive therapy, cardiovascular disease, and bisphosphonate use).
• Contraindications to doxycycline use.
• Smoker status.
• Pregnancy.

### 2.2. Periodontal Parameters

After baseline screening (clinical and radiographic) and diagnosed with periodontitis, patients who met the inclusion and exclusion criteria, according to our internal protocol, were treated as described below. Informed consent was obtained from all subjects involved in this paper. The study was conducted in accordance with the Declaration of Helsinki. All clinical procedures performed in this case series, including incremental ultrasonic instrumentation and local 14% doxycycline (Ligosan), represent the department′s standard of care protocol for the management of deep infrabony defects in aesthetic areas, applied independently of any research intent. No randomization, experimental allocation, or deviation from standard clinical decision‐making was introduced, and all clinical and radiographic parameters are routinely assessed during standard periodontal care regardless of study participation.

The same operator (D.B.) collected the following parameters at two different times: at baseline and 3 months after nonsurgical therapy:

• The full‐mouth plaque score (FMPS) represents the percentage of total surfaces (four aspects per tooth) that reveal the presence of plaque out of the patient′s total tooth surfaces.
• The BOP is recorded dichotomously using a periodontal probe with a force of about 0.3 N, yielding a positive or negative result.
• The full‐mouth bleeding score (FMBS) represents the percentage of surfaces (four aspects per tooth) with BOP+ out of the patient′s total tooth surfaces.

At the level of the site with the infraosseous defect, specifically at the deepest probing point at the mesial or distal aspect overlying the defect, the following values were measured [6]:

• PD: distance between the gingival margin and the probe tip.
• REC: gingival REC, distance between the cement‐enamel junction (CEJ) and the gingival margin.
• CAL: distance between the CEJ and the probe tip.
• BOP.

**Figure 3:**
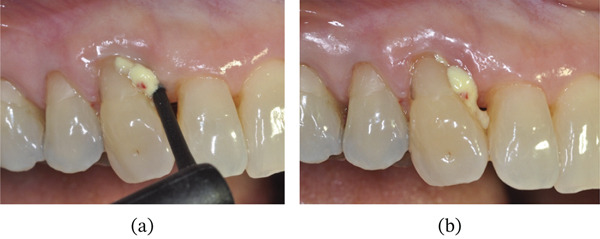
(a, b) Application of local antibiotic (doxycycline 14%) at the level of the site with an infraosseus defect.

**Figure 4:**
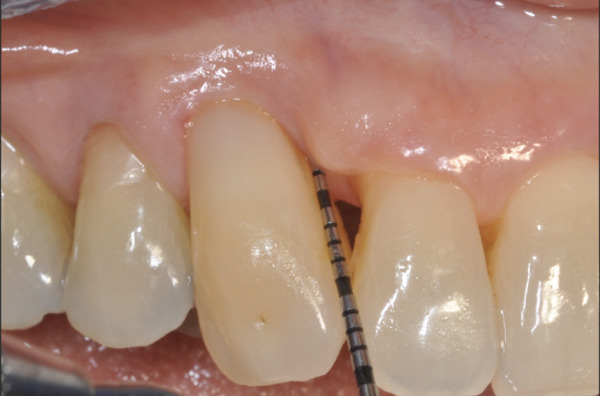
Buccal aspect at the level of the site with an infraosseus defect 3 months after the nonsurgical therapy.

All measurements were taken by using a pressure‐sensitive hand probe and were approximated to the nearest millimeter (Figures 1 and 2). All PD, REC, and CAL measurements were referenced to the anatomical CEJ. Where a pre‐existing cervical restoration was present (as in the case illustrated in Figures 1, 2, 3, and 4), measurements were consistently referenced to the original anatomical CEJ, which remained clearly identifiable at both time points.

### 2.3. Nonsurgical Periodontal Therapy

Nonsurgical periodontal therapy was conducted by the same operator (I.M.) throughout the oral cavity with supragingival and subgingival scaling and root planing with a quadrant approach in two or four sessions.

The etiological therapy is aimed at reducing periodontal infection before proceeding with regenerative periodontal surgery for the infraosseous defect. The primary objectives of this therapy were to lower the FMPS and FMBS to below 20% and to decrease the PD. Surgical therapy was only scheduled for sites where these procedures did not result in a reduction of PD at re‐evaluation at 3 months. No regenerative periodontal surgery would be planned until the FMPS and FMBS were both reduced to less than 20%.

### 2.4. Nonsurgical Periodontal Therapy at the Level of the Infrabony Defect, With a Minimally Invasive Approach

At the site of the infrabony defect, a sequence of ultrasonic tips was used as follows: First, Tip A was inserted subgingivally to a maximum depth of 4 mm; next, Tip P was utilized from 4 to 7 mm; and finally, Tip PS was used from 7 to 11 mm subgingivally. These tips have a progressively decreasing diameter along their length, which is comparable with that of a periodontal probe. Therefore, the soft tissue trauma caused by ultrasonic instrumentation should not exceed that of periodontal probing. In contrast, the use of curettes is not recommended, as it may lead to unintentional soft tissue curettage during the procedure.

At the end of the etiological therapy, a 14% doxycycline topical antibiotic^§^ was then applied locally, starting from the bottom of the pocket, carried by a biodegradable carrier that allows its controlled release (*Ligosan, Kulzer*). The gel was delivered using the dedicated application cannula, filling the pocket from the base until the material was visible at the gingival margin, consistent with the manufacturer′s instructions. Postoperative instructions for patients included avoidance of interdental cleaning at the treated site for 10 days following each application, avoidance of hard food in the treated area, continuation of gentle buccal tooth brushing, and avoidance of alcohol‐containing mouthrinse for the first week after each application. A second application of the same antibiotic was inserted into the pocket after 7 days *(*Figure 3a,b). The rationale for the second application was to extend local doxycycline concentrations beyond the approximate 7–10‐day biodegradation half‐life of the carrier, maintaining therapeutic levels during the critical early healing window.

### 2.5. Re‐Evaluation Visits

One month after completing nonsurgical therapy, patients were scheduled for a follow‐up appointment to assess their adherence to home oral hygiene routines. During these visits, personalized motivation and detailed oral hygiene instructions were reinforced as needed to assure optimal compliance. At the 3‐month mark after nonsurgical therapy, all patients underwent an extensive clinical examination, during which the previously mentioned clinical parameters were recorded to evaluate treatment outcomes and overall improvements in oral health *(*Figure 4).

### 2.6. Statistical Analysis

Given the descriptive and exploratory nature of this case series, inferential analyses were considered exploratory. Continuous variables (PD, REC, and CAL) are expressed as mean ± standard deviation (SD). The dichotomous variable BOP is reported as a proportion (number of positive sites out of the total number of treated sites). Within‐group changes between baseline and 3‐month values are presented as mean differences. All measurements were performed at the site level, with one site per patient included in the analysis (*n* = 10). A paired two‐tailed *t*‐test was used to explore differences between baseline and 3‐month values for PD, gingival REC, and CAL. Due to the absence of a control group and the limited sample size, no between‐group comparisons or adjustment for multiple testing were performed. The primary outcome was the change in PD from baseline to the 3‐month re‐evaluation visit; secondary outcomes included changes in REC, CAL, and BOP resolution rate.

## 3. Results

In all treated patients (mean age 57.2 ± 7.5 years, range 44–68; 6 males, 4 females) diagnosed with periodontitis Stage III Grades B–C [18], an improvement in PD value was observed without any REC increase; notably, BOP value was negative in all patients, indicating the suitability for regenerative periodontal surgery.

Table 1 depicts PPD, REC, CAL, and BOP parameters at baseline. At baseline, mean FMPS was 65*%* ± 15*%* and FMBS was 63*%* ± 22*%*. At 3‐month re‐evaluation, both had decreased below 20% in all patients.

**Table 1:** Demographic characteristics and baseline periodontal parameters.

	Gender	Age	Stage, grade	Defect	PD	REC	CAL	BOP
**Patient 1**	M	56	III, B	#13 mesial	8 mm	0 mm	8 mm	+
**Patient 2**	M	49	III, B	#14 distal	10 mm	2 mm	12 mm	+
**Patient 3**	F	51	III, B	#22 distal	8 mm	0 mm	8 mm	+
**Patient 4**	M	44	III, C	#25 mesial	15 mm	1 mm	16 mm	+
**Patient 5**	F	62	III, C	#23 mesial	11 mm	2 mm	13 mm	+
**Patient 6**	F	65	III, B	#14 mesial	10 mm	0 mm	10 mm	+
**Patient 7**	F	56	III, B	#22 distal	9 mm	1 mm	10 mm	+
**Patient 8**	M	68	III, C	#15 mesial	11 mm	2 mm	13 mm	+
**Patient 9**	M	62	III, C	#13 mesial	9 mm	1 mm	10 mm	+
**Patient 10**	M	59	III, B	#14 distal	8 mm	0 mm	8 mm	+

Abbreviations: BOP, bleeding on probing; CAL, clinical attachment level; PPD, probing pocket depth; REC, recession.

Table 2 shows the results obtained at 3 months after etiological therapy with the proposed minimally invasive approach.

**Table 2:** Three months (re‐evaluation visit) PD, REC, CAL, and BOP parameters.

	PD	REC	CAL	BOP
**Patient 1**	4 mm	0 mm	4 mm	—
**Patient 2**	6 mm	1 mm	7 mm	—
**Patient 3**	4 mm	0 mm	4 mm	—
**Patient 4**	9 mm	1 mm	10 mm	—
**Patient 5**	8 mm	1 mm	9 mm	—
**Patient 6**	8 mm	0 mm	8 mm	—
**Patient 7**	7 mm	0 mm	7 mm	—
**Patient 8**	7 mm	1 mm	8 mm	—
**Patient 9**	6 mm	0 mm	6 mm	—
**Patient 10**	6 mm	0 mm	6 mm	—

Abbreviations: BOP, bleeding on probing; CAL, clinical attachment level; PPD, probing pocket depth; REC, recession.

A comparison of the mean values found at baseline and the mean values found at 3 months is reported in Table 3. PD, REC, and CAL (PPD + REC) average were measured at the site with the infraosseous defect. A statistically significant reduction was observed for PD and CAL (*p* < 0.001). Regarding REC, the mean value decreased from 0.9 ± 0.9 mm at baseline to 0.4 ± 0.5 mm at 3 months (mean reduction: 0.5 mm; paired *t*‐test, *t*(9) = 3.00, *p* = 0.015). This reduction was driven by five patients (Patients 2, 5, 7, 8, and 9), each showing a 1‐mm decrease in REC, whereas the remaining five patients showed no change. This reduction likely reflects resolution of gingival edema associated with inflammation control rather than true coronal migration of the gingival margin, and is close to the limit of probing reproducibility. Critically, no patient experienced an increase in gingival REC.

**Table 3:** Comparison of the mean values found at baseline and at 3 months.

	Baseline	3 months
**PD average**	7.3 +/−1.9	5.2 +/−1.3
**REC average**	0.9 ± 0.9	0.4 ± 0.5
**CAL average**	8.0 +/−1.2	5.9 +/−1.4
**BOP**	10/10	0/10

Abbreviations: BOP, bleeding on probing; CAL, clinical attachment level; PPD, probing pocket depth; REC, recession.

## 4. Discussion

The primary objective of regenerative therapy is to reduce PD (PD reduction). Typically, this reduction can be accomplished in two ways: The first method aims for CAL gain, whereas the second method leads to an increase in gingival REC (REC increase). Since the main intent of regenerative therapy is to solely reduce PD to achieve CAL gain, it is essential to avoid gingival REC.

Etiological therapy prior to regenerative surgery is crucial for the success of treatment. The primary goal of this therapy is to resolve periodontal infections throughout the mouth. This should involve achieving a FMPS and FMBS of less than 20%. Additionally, PDs for all teeth should be reduced through careful supragingival scaling and root planing, particularly at sites with PPDs greater than 4 mm.

Regenerative surgery cannot be scheduled until the patient′s FMPS and FMBS are both below 20%. The objective of etiological therapy in areas affected by infrabony defects is not only to completely resolve clinical signs of inflammation but also to minimize the risk of REC in both interdental and buccal soft tissues. Superficial inflammation is addressed through effective supragingival plaque control, aiming for a plaque index of 0% in the surgical area. Meanwhile, deep inflammation, indicated by BOP, is managed by eliminating supragingival plaque and calculus deposits using only ultrasonic instruments.

This clinical case series study evaluated a minimally invasive, nonsurgical protocol for managing deep infrabony defects in aesthetically sensitive areas. By combining subgingival ultrasonic instrumentation with the adjunctive use of locally delivered doxycycline, the proposed approach aimed not only to reduce PDs and clinical inflammation but also to maintain the integrity of the SCST, a crucial determinant of long‐term esthetic outcomes and surgical predictability.

A central finding of this investigation is the lack of an increase in gingival REC following treatment. This result is of particular clinical importance, as conventional nonsurgical therapy, especially when performed with manual curettes, is often associated with an increase in REC due to postinflammatory soft tissue shrinkage and trauma‐induced remodeling [20, 21]. By contrast, in this study, mean REC decreased significantly from 0.9 ± 0.9 mm at baseline to 0.4 ± 0.5 mm at 3 months (*p* = 0.015), and no patient experienced an increase in gingival REC. In five patients, a modest reduction of 1 mm in REC was observed, which is most likely attributable to resolution of gingival edema following inflammation control rather than a true coronal shift of the gingival margin. At this magnitude, the change is also close to the limit of probing reproducibility, and statistical significance should not be overinterpreted as a confirmed biological effect. This outcome may be attributed in part to the careful use of ultrasonic tips [6], with decreased incremental diameters, which allow efficient debridement with minimal disruption to the epithelial attachment and connective tissue architecture. Ultrasonic instrumentation, when delivered with precision and reduced power settings, has been shown to be less traumatic to the gingival tissues compared with manual scaling [21, 22]. However, these findings should be read with caution given the uncontrolled design, small sample, and absence of a comparator group. Another key factor that may have contributed to soft tissue stability is, as a biologically plausible hypothesis, the topical application of doxycycline (*Ligosan*). Beyond its antimicrobial activity, doxycycline exhibits MMP inhibitory effects, which are known to reduce connective tissue breakdown and promote wound stability [16, 17]. MMPs, particularly MMP‐8 and MMP‐9, are significantly elevated in periodontal disease and contribute to extracellular matrix degradation. By inhibiting MMP activity, doxycycline may help stabilize the soft tissue extracellular matrix, potentially limiting apical migration of the junctional epithelium and preserving the gingival margin during the early healing phase [16, 17]. However, the present study did not directly evaluate these molecular pathways, and their contribution to the observed clinical outcomes cannot be established from these data alone; only speculative statements can be made.

Concerning the systemic safety profile of the local doxycycline application, plasma concentrations following subgingival delivery of Ligosan have been shown to remain substantially below the threshold required for systemic antimicrobial activity, confirming that systemic exposure is negligible [13, 15]. Locally, the short‐duration two‐dose protocol and the controlled‐release biodegradable carrier further minimize cumulative tissue exposure. Regarding antibiotic resistance, the supratherapeutic local concentrations achieved with pocket delivery are considered to exert less selective pressure for resistance than systemic subtherapeutic dosing. Nevertheless, antibiotic stewardship principles must be respected, and the adjunctive use of local antibiotics should be reserved for appropriately selected cases as recommended by current EFP guidelines [11, 12]. Future studies should include microbiological assessments to monitor resistance patterns.

Furthermore, doxycycline has been shown to possess anti‐inflammatory and angiogenic‐modulating properties, contributing to enhanced soft tissue wound healing and modulation of the local inflammatory environment [17, 23]. These effects may favor the resolution of inflammation without triggering REC, especially in the context of delicate supracrestal tissues in aesthetic areas.

Importantly, all treated sites in this study showed complete resolution of BOP, which is consistent with previous findings supporting the effectiveness of locally delivered doxycycline as an adjunct in nonsurgical periodontal therapy [24]. When combined with gentle ultrasonic debridement, this approach resulted in significant PD reductions (mean reduction: 2.1 mm) and CAL gains (mean gain: 2.1 mm) while maintaining the soft tissue margin—a particularly challenging goal in deep defects within esthetic regions.

The stability of the gingival margin observed herein stands in contrast to traditional outcomes, where posttherapy REC is often considered a trade‐off for inflammation control [25]. Our findings support the hypothesis that modulation of the wound environment through doxycycline application, along with tissue‐sparing instrumentation, may represent a novel strategy for soft tissue preservation in the presurgical phase. It must be acknowledged, however, that because full‐mouth nonsurgical therapy was performed concurrently with the target site treatment, general improvement in gingival health may have contributed to tissue stabilization independently of the specific doxycycline protocol. This potential confounding factor cannot be excluded in the absence of a controlled comparator group.

Additionally, these findings highlight the importance of initial SCST integrity, which likely played a synergistic role in preserving soft tissue height. Previous studies have confirmed that thicker SCST phenotypes are more resilient to mechanical insult and exhibit better regenerative potential postsurgery [26–28]. The present results reinforce this principle and suggest that enhancing or preserving SCST before regenerative surgery may, as a working potential hypothesis, increase the likelihood of optimal regenerative outcomes, a proposition that requires validation in future studies incorporating a surgical phase and appropriate regenerative outcome measures.

In contrast, a purely nonsurgical approach has been described by Nibali et al. [29]. In this method, when an infraosseous vertical defect is present, nonsurgical periodontal therapy is performed using ultrasonic tips and Gracey curettes. After the treatment, the patient is re‐evaluated after 12 months. The authors report observing a partial “bone fill” radiographically with nonsurgical periodontal therapy alone. However, although the reduction in PPD is noted, it is accompanied by an increase in REC, which is particularly undesirable in the esthetic region. Moreover, Nibali and Cortellini [30] in 2025 suggested that alveolar bone changes occur following nonsurgical periodontal therapy, particularly in infrabony defects, proposing changes in current clinical protocols.

In conclusion, the minimally invasive, nonsurgical protocol evaluated in this study appears to preserve the soft tissue margins while achieving substantial improvements in PD and CAL. The adjunctive use of locally delivered doxycycline, through its antimicrobial, anti‐inflammatory, and MMP‐inhibitory effects, may, as a plausible biological hypothesis requiring further validation, offer an additional layer of protection for the delicate gingival architecture, especially in esthetic areas. These promising results merit further validation in controlled clinical trials with histological correlation and long‐term esthetic evaluation.

This study has presented some limitations, including a small sample size and the absence of a randomized control group. The follow‐up period of 3 months, although sufficient to assess initial healing and tissue response, does not provide insight into long‐term stability or postsurgical regenerative outcomes. Furthermore, the modified two‐application doxycycline protocol has not been evaluated in a controlled trial; the potential confounding effect of concurrent full‐mouth periodontal therapy on soft tissue stability cannot be excluded; and all statements regarding improved surgical predictability or regenerative potential must be interpreted as speculative hypotheses pending validation in adequately powered RCTs with a regenerative surgical endpoint. Future studies should incorporate volumetric assessment of SCST using digital or CBCT‐based techniques, histologic validation of soft tissue preservation, and patient‐centered esthetic outcome measures. Additionally, randomized comparisons with manual instrumentation and without local adjuncts are warranted to isolate the effects of each protocol component.

## 5. Conclusions

Nonsurgical periodontal therapy combining incremental ultrasonic instrumentation with adjunctive locally delivered doxycycline (14%) demonstrated the ability to reduce PD and resolve BOP without inducing gingival REC; a small, statistically detectable reduction in REC was observed in half of the patients, of uncertain clinical significance given measurement‐level constraints. This tissue‐sparing approach proved suitable across multiple infrabony defects in the aesthetic zone, including sites with adjacent buccal RECs, without requiring space‐maintaining biomaterials. By achieving adequate infection control without iatrogenic REC, the protocol may optimize, as a working hypothesis, soft‐tissue architecture prior to regenerative surgery; whether this translates into improved predictability of aesthetic outcomes remains to be tested. These outcomes are preliminary and require confirmation through adequately powered RCT.

## Author Contributions

Ilham Mounssif: conceptualization, investigation, writing—original draft preparation, and writing—review and editing. Diego Bianchelli: conceptualization, investigation, writing—original draft preparation, and writing—review and editing. Claudio Mazzotti: investigation and writing—review and editing. Valentina Bentivogli: investigation and writing—review and editing. Anna Skurska: writing—review and editing. Martina Stefanini: conceptualization, supervision, and writing—review and editing.

## Funding

No funding was received for this manuscript. Open access publishing facilitated by Universita di Bologna, as part of the Wiley ‐ CRUI‐CARE agreement.

## Disclosure

All authors have read and approved the final version of the manuscript. The corresponding author had full access to all of the data in this study and takes complete responsibility for the integrity of the data and the accuracy of the data analysis.

## Consent

Informed consent was obtained from all subjects involved in the study.

## Conflicts of Interest

The authors declare no conflicts of interest.

## Endnotes

^§^14% doxycycline topical antibiotic is a standard of care in the Department.

## Data Availability

The data that support the findings of this study are available on request from the corresponding author. The data are not publicly available due to privacy or ethical restrictions.
